# Early sowing can boost grain production by reducing weed infestation in organic no‐till wheat

**DOI:** 10.1002/jsfa.11973

**Published:** 2022-05-17

**Authors:** Rosolino Ingraffia, Gaetano Amato, Paolo Ruisi, Dario Giambalvo, Alfonso S Frenda

**Affiliations:** ^1^ Department of Agricultural, Food and Forest Sciences University of Palermo Palermo Italy

**Keywords:** Mediterranean environment, no tillage, organic farming, *Triticum durum*, weed flora composition

## Abstract

**BACKGROUND:**

Conservative tillage techniques have several agro‐ecological benefits for organic farming. The application of these techniques, however, can create quite a few challenges due to the increased weed competition. Here, we report the results of an organic field experiment in which the responses of wheat and weeds to no tillage (NT) were evaluated compared with conventional tillage (CT). We also tested the hypothesis that, under NT, moving up the sowing date, compared with using the ordinary sowing date for the study area, can result in increased competitiveness of the crop against weeds. Two wheat genotypes, a modern variety and an ancient landrace, were tested.

**RESULTS:**

Substantial reductions in grain yield and protein content were observed in wheat under NT than under CT when the ordinary sowing date was used. This was mainly due to the considerable increase in weed biomass under NT. The tillage system also altered the composition of weed flora, with some species favored under NT and others under CT. In general, early sowing mitigated the detrimental effect of NT on yield. The two genotypes responded differently to the treatments. The early sowing in the modern variety reduced but did not eliminate the advantages of CT over NT, whereas no appreciable differences in grain yield were observed between CT and NT in the landrace.

**CONCLUSION:**

Our results show clearly that, under organic management, using NT alone as a substitute for CT is not agronomically feasible. Moving up the sowing date and using a competitive genotype can help mitigate the negative effects of NT, but surely a more effective application of NT could be achieved by acting simultaneously on other factors of the cropping management system (e.g. crop rotation, fertilization strategy, type of seeder). © 2022 The Authors. *Journal of The Science of Food and Agriculture* published by John Wiley & Sons Ltd on behalf of Society of Chemical Industry.

## INTRODUCTION

Organic farming practices usually involve conventional tillage (CT) with deep soil inversion by moldboard plowing (followed by secondary tillage) to incorporate crop residues and organic fertilizers into the soil (which speeds up their mineralization and thus increases the release of nutrients to the crop), suppress soilborne pathogens and disease, and control weeds.^1^, ^2^ This latter effect is achieved by burying both the weed seeds and the existing weed flora deep in the soil through soil inversion, as well as by mechanically killing the weed plants that gradually emerge prior to crop sowing through secondary tillage.^3^, ^4^ Such actions often have benefits in terms of crop growth and yield under organic management. However, intensive tillage is not without its drawbacks, which generally include depleted soil organic matter, deteriorating soil structure, increased risk of soil erosion, and reduced soil biological activity and biodiversity, which lead progressively to a decline in soil quality and fertility, and hence to soil degradation.^5^, ^6^, ^7^, ^8^ Such effects run contrary to a key principle of organic farming, which is to preserve and improve the quality and functioning of the soil.

Integrating a form of conservation tillage into organic farming might alleviate some of the negative consequences of continuous CT, thus preventing soil degradation.^9^, ^10^ The term ‘conservation tillage’ encompasses a variety of non‐inversion tillage techniques that imply shallow working depths (e.g. reduced tillage with tines or discs) or even the omission of any type of soil tillage at all (i.e. no tillage (NT)). There is abundant evidence that, compared with CT, NT provides various ecological benefits. For example, it prevents soil erosion,^7^ improves many components of soil fertility (e.g. organic matter content, macro‑ and micro‐fauna and microbial activity and diversity^11^, ^12^, ^13^, ^14^), and reduces fuel consumption and carbon dioxide emissions.^15^, ^16^ This means that adopting this technique might meet many of the basic demands of organic farmers, increasing the sustainability of organic agriculture well beyond the avoidance of synthetic agrochemicals. Nonetheless, the use of organic no‐till systems is still very limited worldwide, and it is not surprising that most farmers perceive the difficulty of achieving effective weed control as the main technical barrier to the adoption of conservation tillage, and in particular NT, in organic systems. Many studies conducted in conventional systems in which the application of NT is associated with the distribution of non‐selective herbicides (e.g. glyphosate) have shown that this technique increases weed infestation and changes the composition of the weed community.^17^, ^18^, ^19^ Research on the application of conservation tillage systems in organic farming is extremely limited and in general has shown that problems relating to weed control are further exacerbated.^20^, ^21^

In the past few decades, in temperate climate zones under humid and cool conditions, some researchers have focused on the use of cover crops (to be terminated before sowing of the cash crop) to increase soil fertility and limit the presence of weeds while protecting the soil against erosion; for example, see Peigné *et al*.^2^ and Vincent‐Caboud *et al.* ^8^, ^17^ These authors have argued that implementing cover crop‐based no‐till systems in semiarid Mediterranean regions is extremely problematic, as cover crops are difficult to establish in water‐limited climates, compete fiercely with cash crops (especially for soil water and nutrients), and are difficult to devitalize in a juvenile phase (the more advanced the stage of development of the plants, the more effective the technique). Certainly, efforts should be made to enable NT in organic farming; this can be achieved by adopting a systemic approach^22^ and attending simultaneously to other factors of crop management. For example, in the Mediterranean environment, moving up the sowing date of autumn/spring crops when most weed plants are still poorly developed would mean the sowing itself would kill many of the weeds. Moreover, sowing in early autumn, when temperatures are still relatively mild, could accelerate initial growth, thus reducing the period during which crops are particularly vulnerable to competition from weeds. It is usually difficult to realize early sowing in CT systems because proper seedbed preparation requires time for clods formed as a result of plowing to be broken down by natural weathering processes and by one or more secondary tillage operations. Furthermore, many organic farmers choose to delay sowing by using the stale seedbed technique to encourage the emergence of weeds, which are then eliminated by harrowing.

Therefore, a field experiment was performed in a semiarid Mediterranean environment (Sicily, Italy) during two consecutive growing seasons, with the overall objective of assessing the feasibility of NT under organic management as an alternative to CT. The specific aim was to test the hypothesis that in a no‐till organic system, moving up the sowing date, compared with using the standard sowing date for the area, would provide an advantage to the crop (durum wheat, *Triticum durum* Desf., in the present study) in terms of growth and grain yield by increasing its competitiveness against weeds. Moreover, we included two durum wheat genotypes (one ancient Sicilian landrace and one modern cultivar) in the experiment to test the hypothesis that genotypes that differ in their morpho‐agronomic traits and level of competitiveness against weeds respond differently to the application of NT under organic management. The insights obtained from this study are expected to contribute to more successful application of conservation agriculture practices within organic systems of Mediterranean regions.

## MATERIALS AND METHODS

### Experimental site

A field experiment was performed under organic management during two growing seasons (2016–2017 and 2017–2018) at the Pietranera farm, which is located about 30 km north of Agrigento (Sicily, Italy; 37° 32′ N, 13° 31′ E; 178 m a.s.l.). The soil is classified as a Vertic Haploxerept, and characteristics of its 0–40 cm layer are as follows: 525 g kg^−1^ clay, 227 g kg^−1^ silt, and 248 g kg^−1^ sand; pH 8.2 (1:2.5 H_2_O); 16.8 g kg^−1^ total carbon (C; Walkley–Black method); and 1.78 g kg^−1^ total nitrogen (N; Kjeldahl method).

The climate is semiarid Mediterranean, with a mean annual rainfall of 564 mm (1985–2015) concentrated mostly during the autumn–winter period (September–February; 73%) and the spring (March–May; 23%). The mean annual evapotranspiration is about 1100 mm (Penman–Monteith method). The dry period occurs from May to September. The mean air temperature is 15.9 °C in autumn, 9.7 °C in winter, and 16.5 °C in spring. The average minimum and maximum annual temperatures are 9.4 °C and 24.0 °C, respectively. The climatic data during the experimental period were collected from a weather station located about 500 m from the experimental site.

### Experimental design and crop management

The experiment was set up as a split‐plot design with four replications. The main plots (140 m^2^ each) were three soil tillage system–sowing date combinations: CT with the ordinary sowing date of durum wheat (CT‐Ord), NT with an ordinary sowing date (NT‐Ord), and NT with an early sowing date (NT‐Early). The subplots were two durum wheat genotypes: cv. Orizzonte, a modern cultivar, released in 2011 and characterized by a short plant stature, early heading and maturity, and high yield potential; and the landrace Scorsonera, an old Sicilian landrace with a tall plant stature, medium–late heading and maturity, and moderate yield potential. The subplots were 70 m^2^ (20 rows, each 20 m long, spaced 0.175 m apart). In both growing seasons, berseem clover (*Trifolium alexandrinum* L.) was the previous crop; before berseem clover had been sown, the soil had always been managed with one shallow harrowing to prepare a proper seedbed.

In both growing seasons, CT consisted of one moldboard plowing to a depth of 0.30 m in the summer (August), followed by one harrowing before planting; NT consisted of sowing by direct drilling. In CT plots, residues of the previous crop were incorporated into the soil, whereas in NT plots they were left on the soil surface to guarantee that soil coverage was always more than 30%. The ordinary sowing date corresponded to the time when organic durum wheat is usually sown in the study area (i.e. mid‐December), whereas the early sowing plots were sown, in both growing seasons, 1 month before the ordinary sowing date (i.e. mid‐November).

Organic N fertilizer (hydrolyzed leather meal Dermazoto N11; 11% N, 40% organic C) was applied immediately before planting to all plots at a rate of 400 kg ha^−1^. In both growing seasons, a very shallow weed harrowing with a spring tine harrow was carried out before planting in all NT plots to eliminate weeds that had emerged early. In particular, one weed harrowing treatment was carried out in the NT‐Early plots (in mid‐November in both years) and two in the NT‐Ord plots (the first the same time as the treatment done in the NT‐Early plots and the second after about 1 month; that is, immediately before planting of the NT‐Ord plots).

Durum wheat was planted at 400 viable seeds m^−2^ with a no‐till seed drill with hoe openers (Sider.Man; Calà Srl, Caltanissetta, Italy) in all tillage treatments; the appropriate adjustments were made to ensure a homogeneous planting depth. Seeds were inoculated with a mixture of *Glomus* spp., *Trichoderma harzianum*, and plant‐growth‐promoting rhizobacteria (Ekoseed Cereals; Green Srl, Ravenna, Italy) at a dose of 200 g per 100 kg of seed. Sowing dates varied by treatment, but in both growing seasons they fell around 20 November for NT‐Early and around 20 December for both NT‐Ord and CT‐Ord. During the crop cycle, no weed control was practiced; therefore, all natural weeds were left free to grow.

In both growing seasons, at seed maturity, two sampling areas of 5.25 m^2^ each (10 rows, each 3 m long, spaced 0.175 m apart) were identified within each subplot. In these areas, the abundance of weed species was assessed using the Braun–Blanquet method (i.e. a combined cover–abundance estimation technique^23^). Then, all wheat and weeds plants within each sampling area were harvested by cutting the aboveground biomass at soil level. The two plant groups (i.e. crop and weeds) were separated and weighed after oven drying at 80 °C for 24 h to assess their respective total aboveground biomass. The number of spikes was counted before threshing to assess wheat grain yield. Thousand‐kernel weight was estimated by weighing two sets of 250 kernels from each subplot and multiplying the mean weight by four. Soil samples from three layers (0–0.15, 0.15–0.30, and 0.30–0.45 m) were collected from each subplot immediately after harvesting of the durum wheat and analyzed for 2 mol L^−1^ potassium chloride‐extractable ammonium‐N and nitrate‐N^24^ with a Bran & Luebbe II AutoAnalyzer (Bran+Luebbe, Norderstedt, Germany) to determine soil residual mineral N. Later, the total N grain content of the durum wheat was determined on grain samples using the Dumas method (flash combustion with an automatic N analyzer; DuMaster D‐480; Büchi Labortechnik, Flawil, Switzerland; AACC method 46‑30^25^). Grain protein content was then calculated as the product of total N grain content × 5.75. Test weight was determined by means of a TM NG humidimeter (Tripette and Renaud – Chopin, Villeneuve‐la‐Garenne, France).

### Calculations and statistical analysis

Before the analysis, the Braun–Blanquet values were transformed into percentages according to Van der Maarel.^26^ Weed communities under the different treatments (tillage system–sowing date combinations and durum wheat genotypes) were compared using the Shannon diversity index *H*_SH_, calculated as follows:

$$H_{\mathrm{SH}}=\sum_{i=1}^{S}P_{i}\left(\ln P_{i}\right)$$

where

$$P_{i}=\frac{N_{i}}{N_{\text{total}}}$$

where *N*_*i*_ is the number of individuals of species *i*, *N*_total_ is the total number of individuals per sampling area, and *S* is the total number of species found.

We analyzed all data in R^27^ using a generalized least‐squares model in the nlme package^28^ with the varIdDent() function to account for heterogeneity of variance. Model residuals were checked for heteroscedasticity and normal distribution.

Data from each growing season were analyzed separately, and the homogeneity of variances was assessed with Bartlett's test before the combined analysis was performed. We used a mixed model to analyze the combined two‐growing season data set, whereby growing season and replications were considered random, and the tillage system–sowing date combinations and durum wheat genotypes were treated as fixed factors.

We compared all response variables of both NT‐Ord and NT‐Early with CT‐Ord using the dabestr package^29^ to calculate effect sizes as unpaired mean differences and generate bias‐corrected and accelerated bootstrapped 95% confidence intervals. We used this combined approach given increasing recognition of the limitations of using only the *P*‐value and to avoid dichotomous cutoffs.^29^, ^30^

## RESULTS

### Climatic conditions

**Figure 1:**
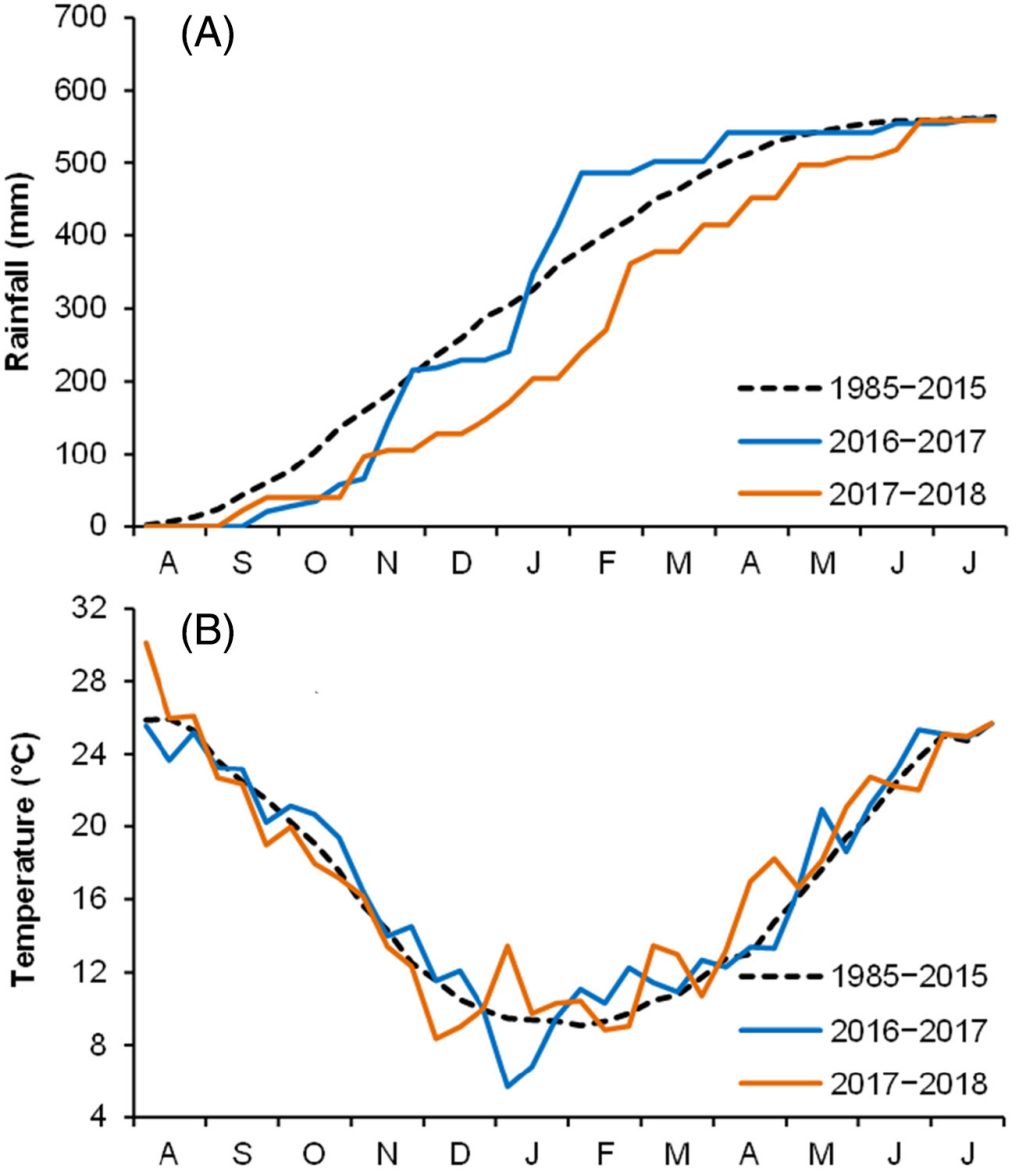
(A) Accumulated rainfall and (B) 10‐day mean air temperature at the experimental site during the two growing seasons (2016–2017 and 2017–2018). The 30‐year average 10‐day temperatures and accumulated rainfall are also included (period 1985–2015).

The total rainfall in the 2016–2017 and 2017–2018 growing seasons (August–July; 561 mm and 569 mm respectively) was almost equal to the long‐term average (Fig. 1(A)). In the first growing season, rainfall mainly occurred in November (158 mm) and between January and the first decade of February (257 mm); it was rather scarce during the spring. In 2017–2018, autumn rainfall was about 45% lower than the long‐term average but spring rainfall was 30% greater. In the two growing seasons, the mean annual temperature was slightly higher (17.0 and 17.1 °C) than the long‐term average (Fig. 1(B)).

### Biomass and grain yields, grain protein content, and soil residual N

**Figure 2:**
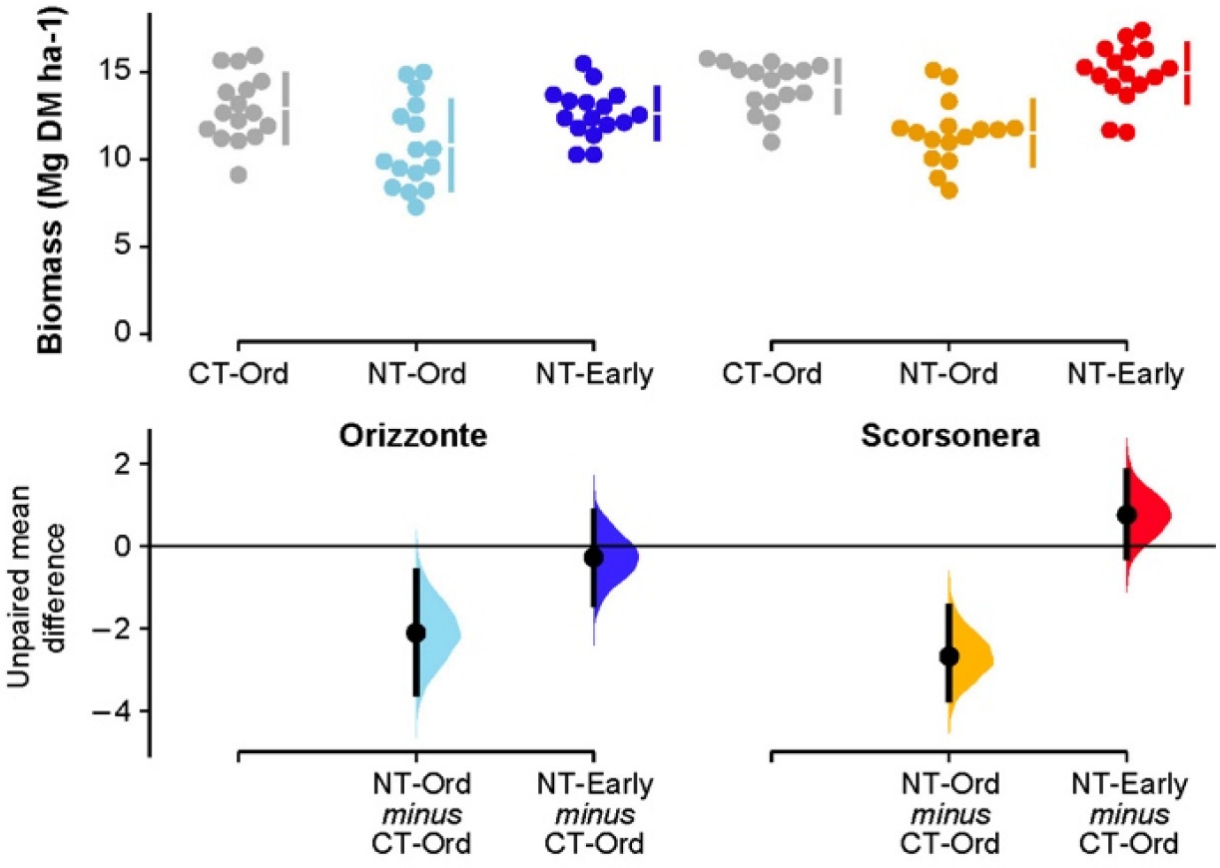
Biomass of durum wheat: raw data (*N* = 16) for the three combinations of tillage system–sowing date displayed separately by genotype (cv. Orizzonte, modern variety; Scorsonera, old landrace). CT‐Ord, conventional tillage–ordinary sowing date (gray dots); NT‐Ord, no tillage–ordinary sowing date (light blue and orange dots); NT‐Early, no tillage–early sowing date (dark blue and red dots). The filled curves indicate the resampled distribution of mean differences Δ for NT‐Ord minus CT‐Ord and for NT‐Early minus CT‐Ord, displayed separately by genotype. Horizontally aligned with the mean of the test group, Δ is indicated by the black circles. The 95% confidence interval of each Δ is illustrated by the black vertical line.

The two durum wheat genotypes varied greatly in total aboveground biomass (on average 12.1 Mg ha^−1^ and 13.5 Mg ha^−1^ for cv. Orizzonte and the landrace Scorsonera respectively; Table 1, Fig. 2), with similar responses by tillage system–sowing date combination. On average, biomass yield was significantly higher under CT than under NT when the ordinary sowing date was used (+21%). In the NT systems, early sowing significantly increased biomass yield by 24% on average compared with the ordinary sowing date (13.8 Mg ha^−1^ and 11.2 Mg ha^−1^ respectively in NT‐Early and NT‐Ord). Moreover, NT‐Early had biomass yields similar to those obtained in CT‐Ord.

**Table 1:** Analysis of variance: *P*‐values for the effects of the applied treatments (tillage system–sowing date and genotype) on the measured traits

Trait	*P*‐value		
	Tillage system–sowing date (T)	Genotype (G)	T × G
Aboveground biomass	0.003	<0.001	0.088
Grain yield	0.008	<0.001	0.011
Spikes m^−2^	0.742	0.717	0.060
Kernels per spike	<0.001	<0.001	0.001
1000‐kernel weight	0.275	<0.001	0.156
Test weight	0.008	<0.001	0.029
Grain protein content	<0.001	<0.001	0.072
Weed biomass	<0.001	<0.001	0.122
Soil residual nitrogen	0.030	0.013	0.260

**Figure 3:**
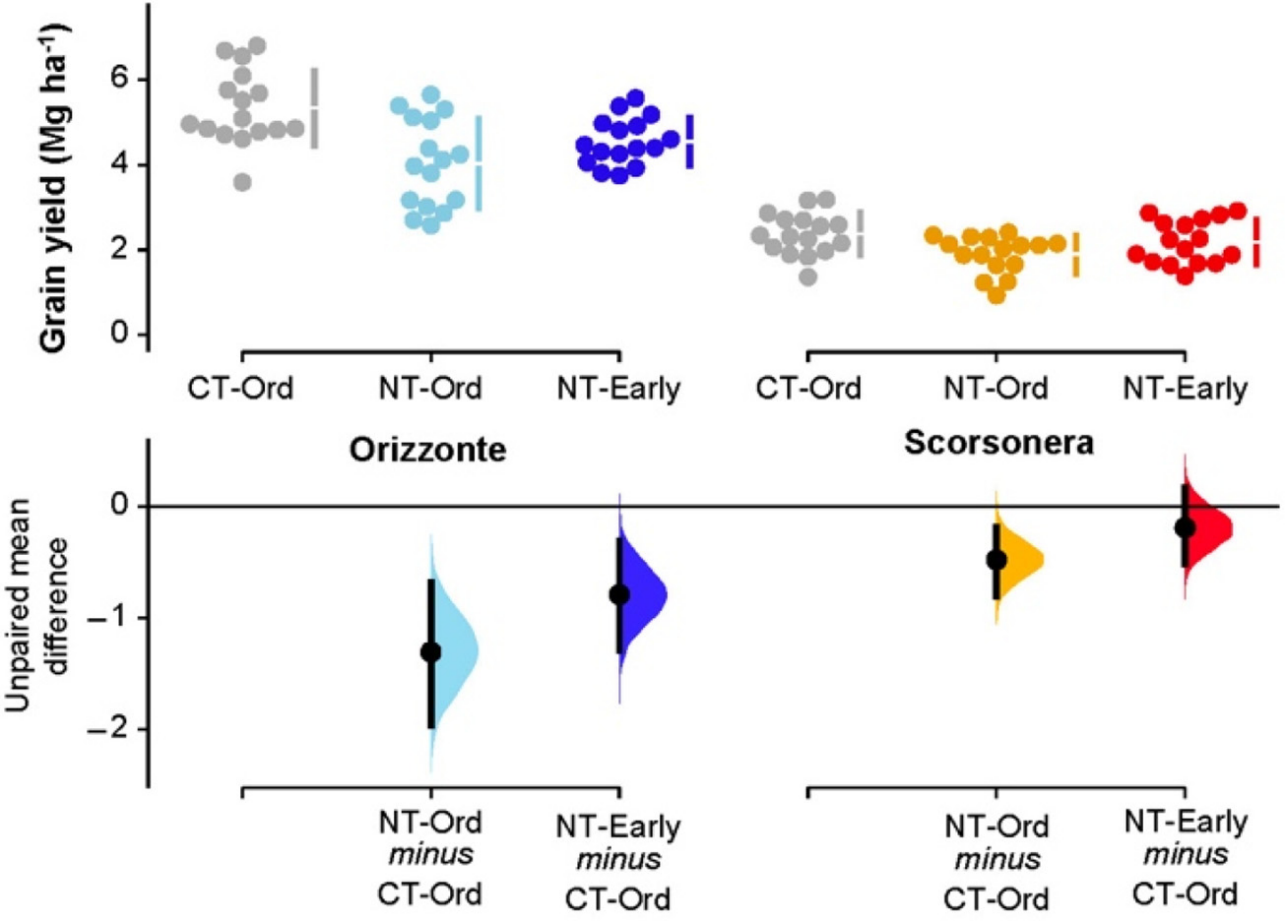
Grain yield of durum wheat: raw data (*N* = 16) for the three combinations of tillage system–sowing date displayed separately by genotype (cv. Orizzonte, modern variety; Scorsonera, old landrace). CT‐Ord, conventional tillage–ordinary sowing date (gray dots); NT‐Ord, no tillage–ordinary sowing date (light blue and orange dots); NT‐Early, no tillage–early sowing date (dark blue and red dots). The filled curves indicate the resampled distribution of mean differences Δ for NT‐Ord minus CT‐Ord and for NT‐Early minus CT‐Ord, displayed separately by genotype. Horizontally aligned with the mean of the test group, Δ is indicated by the black circles. The 95% confidence interval of each Δ is illustrated by the black vertical line.

On average, the grain yield of cv. Orizzonte was considerably higher than that of the landrace Scorsonera (4.64 Mg ha^−1^ and 2.15 Mg ha^−1^ respectively; Fig. 3). The effect of the tillage system–sowing date combination on this trait varied by genotype. In particular, grain yield was significantly higher in CT‐Ord than in NT‐Ord (+32%) in cv. Orizzonte; here, moving up the sowing date reduced but did not cancel out the advantage of CT over NT (5.34 Mg ha^−1^ and 4.55 Mg ha^−1^ respectively in CT‐Ord and NT‐Early). In the landrace Scorsonera, a minor advantage of CT over NT was observed when the ordinary sowing date was used (2.37 Mg ha^−1^ and 1.90 Mg ha^−1^ respectively in CT‐Ord and NT‐Ord), whereas no appreciable difference in grain yield was detected between CT‐Ord and NT‐Early.

**Figure 4:**
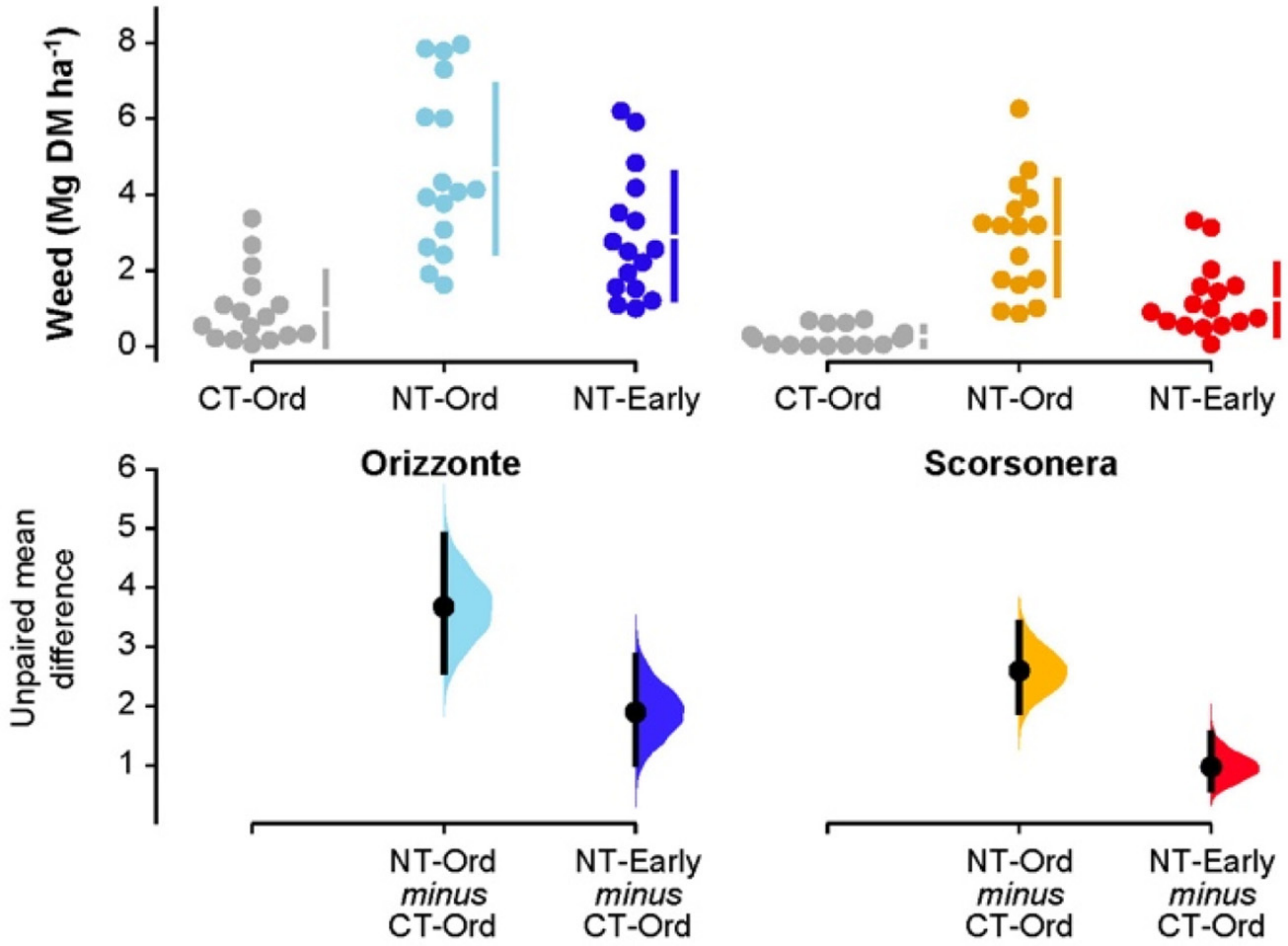
Weed biomass in durum wheat: raw data (*N* = 16) for the three combinations of tillage system–sowing date displayed separately by genotype (cv. Orizzonte, modern variety; Scorsonera, old landrace). CT‐Ord, conventional tillage–ordinary sowing date (gray dots); NT‐Ord, no tillage–ordinary sowing date (light blue and orange dots); NT‐Early, no tillage–early sowing date (dark blue and red dots). The filled curves indicate the resampled distribution of mean differences Δ for NT‐Ord minus CT‐Ord and for NT‐Early minus CT‐Ord, displayed separately by genotype. Horizontally aligned with the mean of the test group, Δ is indicated by the black circles. The 95% confidence interval of each Δ is illustrated by the black vertical line.

With regard to the grain yield components (Tables 1 and 2), no relevant effects of treatment were found for the number of spikes per square meter. On average, the number of kernels per spike was considerably higher in cv. Orizzonte than in the landrace Scorsonera (29.4 and 17.1 respectively) and in CT compared with NT‐Ord, with more marked effects in cv. Orizzonte than in Scorsonera. Moving up the sowing date only made it possible to mitigate the negative effects induced by NT. The 1000‐kernel weight was influenced by genotype alone, being on average 25% higher in cv. Orizzonte than Scorsonera. Great differences in grain protein content were observed between the two genotypes (on average 13.1 g hg^−1^ and 15.1 g hg^−1^ for cv. Orizzonte and Scorsonera respectively; Fig. 4). Moreover, although no appreciable differences were observed among the three treatments in cv. Orizzonte, the grain protein content of Scorsonera was higher in CT‐Ord than in the other two systems.

**Table 2:** Grain yield components, test weight, and soil residual nitrogen (N) measured in the two durum wheat genotypes (cv. Orizzonte, modern variety; Scorsonera, old landrace) under the three combinations of tillage system–sowing date

Treatment	Spikes m^−2^	Kernels per spike	1000‐kernel weight (g)	Test weight (kg hl^−1^)	Soil residual N (mg kg^−1^)
cv. Orizzonte					
CT‐Ord	277	34.5	56.0	80.9	9.6
NT‐Ord	296	24.3*	56.1	81.3	8.7
NT‐Early	278	29.4*	56.9*	80.6	9.6
Scorsonera					
CT‐Ord	275	19.2	45.6	78.2	12.0
NT‐Ord	278	15.6*	44.5*	79.6	9.8*
NT‐Early	292	16.6*	45.3	78.5	10.7
*Means*					
CT‐Ord	276	26.9	50.8	79.5	10.8
NT‐Ord	287	19.9	50.3	80.5	9.3
NT‐Early	285	23.0	51.1	79.6	10.1
cv. Orizzonte	284	29.4	56.3	80.9	9.3
Scorsonera	282	17.1	45.1	78.8	10.9

Abbreviations: CT‐Ord, conventional tillage–ordinary sowing date; NT‐Ord, no tillage–ordinary sowing date; NT‐Early, no tillage–early sowing date.

* Denotes a significant difference (*P* = 0.05) with respect to CT‐Ord.

Soil residual N varied slightly by tillage system–sowing date combination (9.3–10.8 mg kg^−1^ of soil; Table 2). Also, this trait was influenced by genotype, being on average 17% higher in Scorsonera than in cv. Orizzonte (10.9 *versus* 9.3 mg kg^−1^ of soil on average).

### Weed biomass and composition

**Figure 5:**
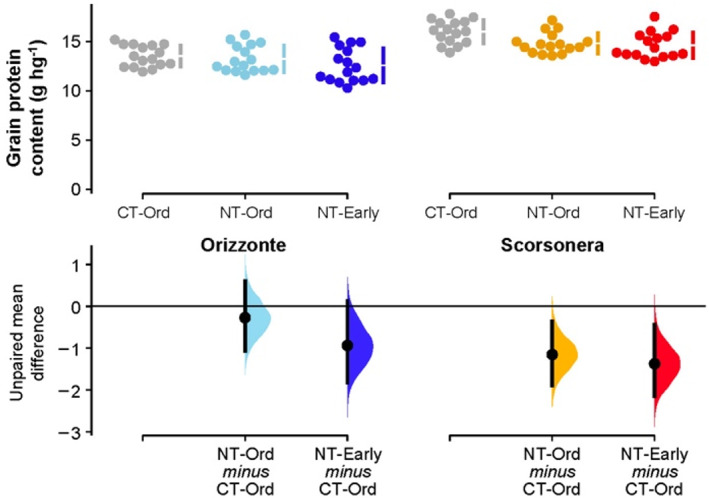
Grain protein content of durum wheat: raw data (*N* = 16) for the three combinations of tillage system–sowing date displayed separately by genotype (cv. Orizzonte, modern variety; Scorsonera, old landrace). CT‐Ord, conventional tillage–ordinary sowing date (gray dots); NT‐Ord, no tillage–ordinary sowing date (light blue and orange dots); NT‐Early, no tillage–early sowing date (dark blue and red dots). The filled curves indicate the resampled distribution of mean differences Δ for NT‐Ord minus CT‐Ord and for NT‐Early minus CT‐Ord, displayed separately by genotype. Horizontally aligned with the mean of the test group, Δ is indicated by the black circles. The 95% confidence interval of each Δ is illustrated by the black vertical line.

Weed biomass differed markedly between the two durum wheat genotypes (on average 2.86 Mg ha^−1^ and 1.46 Mg ha^−1^ in cv. Orizzonte and the landrace Scorsonera respectively; Fig. 5); no ‘tillage system × genotype’ interaction was found for weed biomass. On average, weed biomass was 83% lower under CT than under NT when the ordinary sowing date was used (0.63 Mg ha^−1^ and 3.77 Mg ha^−1^ respectively in CT‐Ord and NT‐Ord). In the NT systems, early sowing greatly decreased weed biomass by 45% on average compared with the ordinary sowing date; nonetheless, the differences between NT‐Early and CT‐Ord systems remained considerable.

More than 20 broadleaf and grass species were found across the six cropping systems (three tillage system–sowing date combinations × two durum wheat genotypes). The mean abundance of the most frequently occurring weeds is reported in Table 3. *Picris echioides*, *Chenopodium* spp., *Ridolfia segetum*, *Sinapis arvensis*, *Lactuca serriola*, *Cichorium intybus*, *Sonchus* spp., and *Phalaris* spp. were the most abundant species. The abundance of many species varied markedly by tillage system–sowing date combination. When the ordinary sowing date was used, the incidence of *Chenopodium* spp., *C. intybus*, *P. echioides*, *L. serriola*, *Sonchus* spp., and *Phalaris* spp. was higher under NT than under CT (the latter three species only with cv. Orizzonte). In contrast, *R. segetum* and *S. arvensis* were more abundant under CT than under NT. In the NT systems, early sowing decreased the incidence of *C. intybus* and *P. echioides* (however, the abundance of this latter species remained higher in NT‐Early than in CT‐Ord) and increased the incidence of *L. serriola*. No effect of moving up the sowing date was observed on the abundance of *Chenopodium* spp., *R. segetum*, or *S. arvensis*. On the whole, the abundance of weeds was markedly higher with the modern cv. Orizzonte than with the landrace Scorsonera.

**Table 3:** Mean abundance per plot of the most frequently occurring weed species and Shannon's diversity index for weeds for the two durum wheat genotypes (cv. Orizzonte, modern variety; Scorsonera, old landrace) under the three combinations of tillage system–sowing date

Weed species	Common name	cv. Orizzonte			Scorsonera			*P*‐value		
		CT‐Ord	NT‐Ord	NT‐Early	CT‐Ord	NT‐Ord	NT‐Early	Tillage system–sowing date (T)	Genotype (G)	T × G
*Avena* spp.	Wild oats	0.06	0.44	0.00	0.00	0.06	0.06	0.391	0.205	0.178
*Carduus* spp.	Thistles	0.25	0.19	0.13	0.06	0.44	0.25	0.564	0.615	0.336
*Chenopodium* spp.	Goosefoots	0.50	5.81*	6.16*	0.38	4.44*	5.84*	0.065	0.550	0.861
*Cichorium intybus*	Common chicory	0.38	3.19*	1.13*	0.00	1.38*	0.13	0.158	0.040	0.515
*Convolvulus arvensis*	Field bindweed	0.06	0.06	0.06	0.00	0.13	0.06	0.715	1.000	0.537
*Kickxia spuria*	Round‐leaved fluellen	2.13	0.38	0.31	1.63	0.31	0.19	0.415	0.677	0.940
*Lactuca serriola*	Prickly lettuce	0.25	2.50*	5.81*	0.13	0.44	1.13*	0.166	0.007	0.083
*Malva sylvestris*	Common mallow	0.00	0.00	0.13	0.00	0.31	0.00	0.510	0.565	0.241
*Papaver rhoeas*	Corn poppy	0.06	0.13	0.06	0.19	0.00	0.06	0.566	1.000	0.177
*Phalaris* spp.	Canary grasses	0.50	3.44*	0.44	0.00	0.06	0.13	0.057	0.010	0.034
*Picris echioides*	Bristly oxtongue	0.38	9.00*	6.84*	0.50	5.19*	1.38*	0.003	0.015	0.164
*Polygonum aviculare*	Prostrate knotweed	0.13	0.13	0.00	0.19	0.00	0.00	0.214	0.631	0.204
*Ridolfia segetum*	False fennel	7.88	2.94*	2.19*	3.56	2.50	1.44*	0.025	0.051	0.170
*Rumex crispus*	Curly dock	0.06	0.00	0.06	0.00	0.00	0.00	0.607	0.155	0.598
*Sinapis arvensis*	Wild mustard	9.34	0.56*	1.44*	3.63	0.19*	0.44*	0.093	0.037	0.107
*Sonchus* spp.	Sow thistles	0.31	5.44*	1.06*	0.25	0.13	0.25	0.054	0.014	0.023
*Trifolium* spp.	Clovers	0.50	1.69*	0.75	0.19	0.94*	0.44	0.024	0.005	0.432
Other species	—	0.00	1.13*	2.13*	0.00	1.44*	0.56*	0.034	0.281	0.109
Shannon's diversity index	—	0.492	0.696*	0.625*	0.347	0.544*	0.545*	0.006	<0.001	0.519

Abbreviations: CT‐Ord, conventional tillage–ordinary sowing date; NT‐Ord, no tillage–ordinary sowing date; NT‐Early, no tillage–early sowing date.

* Denotes a significant difference (*P* = 0.05) with respect to CT‐Ord.

On average, weed diversity (estimated using Shannon's diversity index) was significantly higher under the NT systems (both NT‐Ord and NT‐Early) than under CT for both wheat genotypes (Table 3).

## DISCUSSION

This study advances knowledge of the feasibility of NT within Mediterranean cereal‐based organic cropping systems. This subject is of great interest to Mediterranean organic farmers who are seeking solutions to improve the sustainability of their cropping systems beyond the standards of organic certification. Nevertheless, there has been little investigation of this topic so far, although many tillage studies performed under conventional farming have broadly demonstrated the potential of NT to provide various benefits in Mediterranean areas, both agronomic (higher crop yields than CT, in particular in dry years^12^, ^31^, ^32^, ^33^) and ecological (e.g. prevention of soil erosion, improved soil biological fertility, C sequestration^7^, ^11^, ^14^). Consequently, although the use of NT has been progressively increasing in Mediterranean areas under conventional agriculture in recent years, its adoption within organically managed farming systems is almost nonexistent.

The results of this study show clearly that, under organic management, using NT alone as a substitute for CT, without changing any other agronomic practices in the cropping system, is not agronomically viable. In fact, considerable reductions in both grain yield and protein content were observed in organic wheat under NT‐Ord compared with CT‐Ord (−23% and −5% on average respectively). Such results are largely attributable to the different effects of the two tillage systems on weed growth. In fact, the lack of tillage (i.e. NT) greatly favored weed growth, as highlighted by the values for weed biomass, which were very low under CT‐Ord (0.6 Mg ha^−1^ on average) and very high under NT‐Ord (3.8 Mg ha^−1^ on average). Many studies have shown that the increase in weed pressure under NT is primarily due to the fact that most weed seeds remain on or near the soil surface, which facilitates their germination and seedling emergence.^20^, ^34^ In contrast, soil inversion by moldboard plowing (i.e. CT) distributes weed seeds along the soil profile, most of them deep in the soil, thereby reducing the likelihood of their emergence.^3^, ^35^ Because no direct weed control measures were used during the crop cycle in the present experiment, the more favorable conditions for weed seed germination and growth in NT than in CT resulted in markedly higher weed biomass under NT conditions.

In addition to weed growth, the tillage technique used also influenced the composition of the weed population. In fact, some weeds (in particular *S. arvensis* and *R. segetum*) were favored by the adoption of CT, whereas a considerable increase in other species (e.g. *P. echioides*, *Sonchus* spp., *Chenopodium* spp., *L. serriola*, *C. intybus*, and *Phalaris* spp.) was observed under NT‐Ord compared with CT‐Ord. These results are fully in line with those of previous long‐term tillage experiments conducted under conventional management in the same environment^18^, ^19^ and partly in line with research carried out in other environments.^36^, ^37^, ^38^ According to Alarcón Víllora *et al*.,^39^ changes in weed flora due to changes in the tillage system cannot be attributed to a single cause alone. Rather, many factors drive the composition of the weed community under a given tillage system, including, among others, the timing of seed germination, seed longevity, seed size, seed predation rate (which varies in relation to the allocation of the seed along the soil profile), seed exposure to light, and seed dispersal mechanisms,^40^, ^41^, ^42^ thus contributing to the success of certain species over others.

Data on grain yield components reveal that the competitive effects of weeds on the productivity of the durum wheat began during the stem elongation period. In fact, no differences were observed on average between CT‐Ord and NT‐Ord in the number of spikes per square meter (which is determined during the tillering phase), although considerable differences were found in the number of kernels per spike (+35% in CT‐Ord compared with NT‐Ord on average), which in cereals is mainly determined during the stem elongation phase.^43^ During the grain filling period, the greater weed competition in NT did not result in reductions in the 1000‐kernel weight, and this is probably because of the lower number of kernels per spike observed in NT than in CT, which required a smaller amount of resources for their filling.

The early sowing time mitigated the detrimental effects of NT on the wheat yield response (compared with CT‐Ord, grain yield was −13% in NT‐Early and −23% in NT‐Ord on average). This result is primarily attributable to the reduction in weed biomass, to which several factors may have contributed: (i) the increase in the competitive advantage of the crop over the weeds acquired by a faster initial growth under NT‐Early than NT‐Ord because of more favorable temperatures during the first phases of the biological cycle (i.e. when the seeds of many weeds began to germinate and the seedlings to grow, the wheat plants were more developed and therefore more effective at competing for available resources); (ii) more effective control of weed seedlings during sowing. With regard to this latter factor, it is reasonable to assume that the uprooting of weed seedlings as a result of furrowing (by hoe openers) by the seeder was more effective in NT‐Early than in NT‐Ord because of the smaller size of the weed plants at the time of early sowing. This may explain the considerable reduction observed in *Phalaris* spp., *P. echioides*, *C. intybus*, and *Sonchus* spp., which are annual species with early emergence or perennials with prompt regrowth immediately after the first autumn rains. However, we were surprised to find that moving up the sowing date in NT increased the abundance of *L. serriola* and *S. arvensis* compared with the ordinary sowing time. It is possible that seed germination and seedling emergence of these two species were a little bit delayed compared with the other spontaneous species, so that, when wheat was sown, their plantlets were not yet present in the field. It follows, then, that the effectiveness of early sowing in organic no‐till wheat is, to a certain extent, dependent on the weed flora present. This possibility certainly deserves further investigation.

The two wheat genotypes studied differed widely in terms of grain yield and quality. The modern variety yielded significantly more than the ancient landrace (on average 4.64 Mg ha^−1^ *versus* 2.15 Mg ha^−1^ respectively) despite the latter showing a higher competitive ability against weeds. The clear superiority of the modern variety over the landrace was not entirely expected, given that other research has shown how the advantage of the greater yield potential of modern cultivars is often offset by the higher ability of older genotypes to suppress weeds and use available resources (in particular N) when they are limited and/or contested.^19^, ^44^, ^45^, ^46^ In this study, the higher competitiveness of the landrace Scorsonera over cv. Orizzonte materialized in a strong reduction in weed biomass in all three tillage system–sowing date combinations (−56% in Scorsonera compared with cv. Orizzonte on average). Compared with what was observed in the modern variety, in the landrace the differences in grain yield by tillage system–sowing date were considerably attenuated. This can be explained to a certain extent by both the high capacity of the landrace to tolerate the depletion of resources by weeds^44^, ^47^ and its high ability to suppress weeds, which ensured that, even in the most difficult condition (NT‐Ord), weed biomass did not exceed the threshold beyond which significant variations (reductions) in yield were observed.

It should be emphasized that the ancient genotype excelled in terms of grain protein content (on average 15.1% *versus* 13.1% in Scorsonera and cv. Orizzonte respectively). This is interesting given that durum wheat grown organically is often characterized by a low grain protein content^48^, ^49^ and is often not suitable for use in high‐quality food products.^50^, ^51^ It is also interesting that the protein content of the two genotypes varied in a non‐univocal way with different tillage systems. In particular, no difference emerged for this trait in cv. Orizzonte, whereas grain protein content in Scorsonera was higher in CT‐Ord than in the two NT systems. CT probably increased the availability of N for wheat plants both through greater mineralization^52^, ^53^ and by reducing weed biomass, and therefore weed N removal. This resulted in an increase in grain yield in the modern variety (which is characterized by a very high yield potential) with no significant variation in grain protein content (dilution effect); in contrast, the greater availability of N in CT than in NT was used by the landrace Scorsonera (characterized by a low yield potential) to increase grain protein content but not grain production.

In conclusion, the results of this study confirm that NT does not perform as well as CT in an organic system, mainly due to an increased weed pressure, which decreases yield in both quantitative and qualitative terms. These negative effects take on even greater value if we consider the repercussions on subsequent crops, in which, because of the increase in dissemination by spontaneous species, the difficulty of controlling weeds will be exacerbated. Moving up the sowing time allowed us to mitigate the negative effects of NT by reducing weed density while modifying the composition of the weed flora, disadvantaging some species and favoring others. This suggests that the effectiveness of NT may vary depending on the context in which it is applied. We believe, moreover, that by acting simultaneously on other aspects of the cropping management system (crop rotation, fertilization strategy, type of seeder, plant arrangement pattern, etc.) and trying to enhance positive interactions among the various components of the cropping system, it is possible to identify suitable applications of NT for different agro‐environmental conditions. In this sense, the choice of a suitable genotype is important as well: in this study, cultivating an ancient landrace, tall and with a high competitive ability against weeds, made it possible to drastically limit the weed pressure to reduce differences in grain yield among the different tillage treatments studied. This choice, however, is limited by the low yield that landraces are generally able to supply compared with modern varieties, even if it must take into account the greater quantity of straw produced by the landrace (which is a co‐product that has value) and the qualitative peculiarity of its production,^54^ which certainly represents added value. Finally, the results seem to suggest that, through careful selection, genotypes suitable for NT can be identified that, in an optimized agronomic context, can make it possible to successfully apply NT, certainly an environmentally friendly technique in many respects, in an organic system, even in the Mediterranean environment.
